# Comparison of Two Spectral-Domain Optical Coherence Tomographs for Macular Thickness in Romanian Children Reveals Device-Dependent Normative Data and Significant Gender Differences

**DOI:** 10.3390/diagnostics16040609

**Published:** 2026-02-19

**Authors:** Iulia-Andrada Nemeș-Drăgan, Alexandru Țîpcu, Ana-Maria Drăgan, Raluca Pașcalău, Jennifer Bogdan, Mădălina Claudia Hapca

**Affiliations:** 1 Department of Ophthalmology, “Iuliu Hatieganu” University of Medicine and Pharmacy, 400012 Cluj-Napoca, Romania; madalina.prodan06@gmail.com; 2 Ophthalmology Clinic, Emergency County Hospital, 3-5 Clinicilor Str., 400006 Cluj-Napoca, Romania; 3 Department of Radiotherapy, “Ion Chiricuță” Institute of Oncology, 34-36 Republicii Str., 400015 Cluj-Napoca, Romania; 4 Department of Medicine and Pharmacy, University of Oradea, 410087 Oradea, Romania; 5 General Medicine Section, Faculty of Medicine, “Iuliu Hatieganu” University of Medicine and Pharmacy, 8 Victor Babes Str., 400012 Cluj-Napoca, Romania

**Keywords:** macular thickness, gender differences, normative data, spectral domain OCT, Romanian children

## Abstract

**Objectives**: This study aimed to establish normative data for macular retinal thickness among Romanian children for two different spectral-domain optical coherence tomographs to establish if they are interchangeable and to compare gender-related differences. **Methods**: This analytical prospective study included 226 healthy children aged 3–17. A total of 113 eyes were examined with the Spectralis SD-OCT (Heidelberg Engineering) device and 113 with the Copernicus REVO SD-CT (Optopol Technology). **Results**: Central foveal and average macular thickness was measured and compared. Combining the two groups, measured with both devices, average measurements are as follows. The central foveal thickness was 203.87 ± 19.972 μm in males and 200.84 ± 21.891 μm in females for the left eye and 204.80 ± 21.516 μm and 199.74 ± 22.132 μm, respectively, for the right eye. While central foveal thickness was greater in males, the difference was not statistically significant (*p* = 0.288 for the left eye and *p* = 0.084 for the right eye). The average macular thickness was 256.94 ± 21.566 μm in males and 250.90 ± 23.488 μm in females for the left eye and 258.21 ± 21.30 μm and 249.70 ± 24.706 μm, respectively, for the right eye, showing significantly higher values in males (*p* = 0.048 for the left eye and *p* = 0.007 for the right eye). Significant inter-device variation was also observed across all OCT parameters (*p* < 0.001). **Conclusions**: This study provides normative data for macular thickness in healthy Romanian children for two spectral-domain optical coherence tomographs and may assist clinicians in interpreting paediatric OCT scans more accurately.

## 1. Introduction

Spectral-domain optical coherence tomography (SD-OCT) has emerged as a crucial, noncontact imaging technology in the assessment and management of paediatric retinal diseases. Unlike earlier time-domain OCT (TD-OCT) systems, SD-OCT utilizes a high-speed charge-coupled device camera and applies Fourier transformation to analyse the interference spectrum, which is generated from oscillations proportional to the reflected time delay [1]. This enables higher resolution and faster acquisition speeds, increasing the resolution by up to fivefold and the imaging speed by as much as 60 times compared to TD-OCT [2]. These are critical advantages that facilitate rapid, high-definition imaging, particularly adapted to paediatric populations, where patient cooperation and imaging time can be limiting factors. SD-OCT, particularly the Spectralis system, is especially well suited for the paediatric population, as it incorporates an advanced eye-tracking system that effectively minimizes motion artefacts caused by involuntary eye movements, thereby improving image quality and reproducibility [3]. In contrast, the extended acquisition time required by TD-OCT increases the likelihood of artefacts caused by eye movements [4]. Furthermore, SD-OCT has demonstrated highly reliable and repeatable measurements of macular thickness, showing superior performance compared to earlier TD-OCT systems [5]. The combination of high repeatability, eye-tracking ability, and enhanced resolution enables consistent imaging of the retina over time, supporting longitudinal assessment and allowing clinicians to monitor subtle changes in the macula and detect early pathological alterations that could impact central vision.

Measuring the macular retinal thickness is important in paediatric ophthalmology because it allows for the detection, quantification, and longitudinal monitoring of macular diseases such as macular oedema and developmental and functional macular disorders [6,7], all of which can have a significant negative impact on central vision and visual development in children. Accurate measurement of macular thickness depends on multiple biological and anatomical factors, with gender having a particularly important influence. Numerous studies using SD-OCT have reported that boys tend to have thicker maculae than girls when adjusted for axial length [8,9]. Additionally, variables such as age, refractive status, axial length [10,11], and ethnicity [12] contribute to individual variation, highlighting the importance of considering these factors when interpreting OCT scans. This also underscores the importance of developing sex-specific normative databases to enhance the precision of paediatric retinal assessments. SD-OCT offers clear benefits in paediatric ophthalmology; however, normative paediatric macular thickness databases remain limited [13]. It is therefore crucial to establish age and sex-specific reference values, as the current references are derived from adult and ethnically homogenous populations [14]. The aim of this study is to provide gender-specific normative data on macular thickness for the Romanian paediatric population using two SD-OCT devices, to establish if they are interchangeable and if not, to address the impact of device-specific measurement differences as well as sex-specific and geographical variation in order to optimize paediatric ophthalmic care.

## 2. Materials and Methods

### 2.1. Study Design and Subjects

This study was an analytical prospective one, carried out in two clinics: the Department of Ophthalmology, “Iuliu Hatieganu”, University of Medicine and Pharmacy, Emergency County Hospital in Cluj-Napoca, Romania, and Doctorlens Eye Clinic in Cluj-Napoca, between 1 January 2023 and 31 August 2025. All the investigations in this study were carried out in accordance with the Declaration of Helsinki. Written informed consent was obtained from the parents or legal guardians of the patients for the use of their clinical data and OCT images for research purposes. This study was approved by the Ethics Committee of the “Iuliu Hațieganu” University of Medicine and Pharmacy (135/27 June 2023) and the Institutional Review Board of Cluj County Emergency Clinical Hospital (17733/18 April 2023).

The inclusion criteria included Caucasian children aged 3 to 17 with best-corrected visual acuity (BCVA) of 20/20 on the Snellen visual acuity scale (measured with E charts for children under 7 years old), equal visual acuity between the eyes, with low or medium refractive errors.

The exclusion criteria were the following: prematurity, high refractive error (possibly related to eye structure changes) defined as a spherical equivalent (SE) exceeding ± 6 dioptres (D), history of manifest strabismus or amblyopia, family history of genetic macular disease, chronic medication, delayed psychomotor development, or any other ocular or systemic problems.

All children received comprehensive ophthalmologic evaluations. Best-corrected distance visual acuity (VA) for each eye was assessed using a Snellen chart, both with and without correction. Refractive errors were measured under cycloplegia following the instillation of 1% tropicamide eye drops three times at 10 min intervals, using an auto-refractometer (Topcon KR-8900, Topcon Corporation, Tokyo, Japan). Additional assessments included intraocular pressure measurement, ocular motility evaluation, stereoacuity testing with the Lang test, slit-lamp examination, and dilated fundus examination.

### 2.2. Spectral-Domain Optical Coherence Tomography (OCT) Assessments

Macular imaging was performed using two SD-OCT devices: the Spectralis SD-OCT (Heidelberg Engineering, Heidelberg, Germany) or the Copernicus REVO SD-OCT (Optopol Technology, Zawiercie, Poland) with their respective manufacturer software, operated each by a single experienced technician to ensure consistency. The devices are located in two different outpatient offices, so the patients were examined with to one or the other based on office availability and appointment resulting in a random distribution. None of the patients was examined with both devices. All measurements were acquired using the preselected small eye length setting. For both devices, only well-focused images meeting the manufacturer recommended signal strength criteria were included (Spectralis: >20/40; Copernicus> 7), with the macular measurement area precisely centred and free from alignment errors.

**Figure 1:**
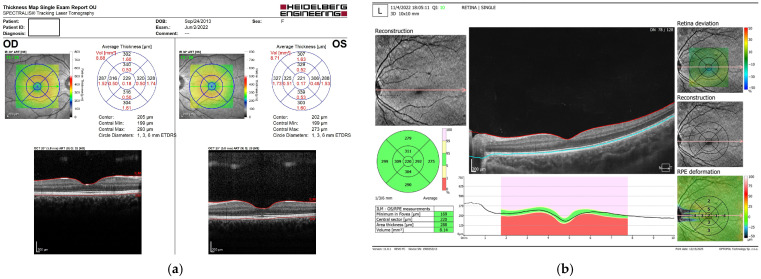
(**a**) Spectralis SD-OCT scan report of macular thickness. (**b**) SOCT Copernicus REVO macular thickness scan report [personal database].

For the Spectralis SD-OCT (version 7.0), examinations were performed after pupillary dilation. The Spectralis SD-OCT system allows adjustable settings to account for variations in eye length. This device uses a superluminescent diode light source (SLED) at 870 nm and acquires 40,000 A-scans per second with an axial tissue resolution of 7 µm. Using the circular scan mode, a 3.45 mm diameter circle was automatically set by the device to measure average retinal thickness. As it is sometimes offset from the foveola, it needs to be moved manually in order to be centred on the foveola. Automatic real-time eye-tracking was activated to reduce noise and motion artifacts, thereby improving image quality by averaging multiple frames of the same location. Macular thickness was automatically measured across standard sectors, as defined by the device software (Figure 1a).

Differences in total retinal thickness measurements between devices were addressed by subtracting the RPE thickness measured on Spectralis from the total retinal thickness. This adjustment was applied when comparing Copernicus REVO and Spectralis measurements, with an expected residual difference of approximately 10–15 μm.

The Copernicus REVO SD-OCT also employs a child friendly real-time eye-tracking system, which accounts for blinking, lapses in fixation, and spontaneous eye movements throughout the OCT scan. This machine uses a superluminescent diode (SLED) light source with a central wavelength of approximately 830 nm, achieving an axial resolution of 5 µm in tissue and an A-scan rate of approximately 130,000 A-scans per second. Automatic macular scanning was used for cooperative participants, after pupillary dilation, with manual scanning applied when necessary and without any incorporated axial length correction. The macula measurements were analysed using the captured OCT images in the appropriate macular scan mode after pupillary dilation. The macular thickness profile represents the thickness along a circular scan centred on the fovea, while the macular grid depicts the thickness across the entire scanned area. The resulting measurements are displayed in a macular thickness map, which includes concentric rings around the fovea to provide sectoral thickness analysis of different macular regions (Figure 1b).

Both devices provided reproducible, high-resolution imaging of the macula, allowing for a quantitative comparison of macular retinal thickness between devices and across study participants; however, neither device had established normative data available.

### 2.3. Statistical Analysis

We reported both counts and percentages for nominal variables, and we compared means, medians, and standard deviations for the continuous variables. We used the Kolmogorov–Smirnov and Shapiro–Wilk tests to assess for distribution types, alongside Q-Q plots and histograms. Different groups were compared using two-tailed independent sample *t*-tests or Mann–Whitney U tests (dependant on distribution type). Categorical variables were assessed for independence using chi-square/exact Fisher testing on the derived crosstabs. Linear relationships were assessed for using Spearman’s correlations and reporting Spearman’s rho. Microsoft Office Excel (Office 365 Suite) was used to create the databases. All statistical analysis was performed using IBM SPSS Statistics v.26.0.0, and 95% confidence intervals are reported as indicated. The threshold of statistical significance was set at a Pearson’s *p* = 0.05.

## 3. Results

### 3.1. Study Cohort

A total of 240 children were included in this study. Fourteen participants were excluded from this study due to poor image quality. A total of 226 subjects met the inclusion criteria, half of which were scanned with Spectralis SD-OCT and the other half with REVO 80 SD-OCT. The mean subject age (± SD) was 8.5 ± 3.5 years (range 3 to 17 years).

### 3.2. Statistical Power of This Study

Given an acceptable target type I error rate (alpha) of 0.05 and a desired power (1 minus beta—type II error rate) of 80%, with a sampling ratio of 1 (equal split between the two devices), we conducted a power analysis targeted at an average MDE (minimum detectable effect) of 10 on the absolute difference between the SD-OCT results given by the two measurement devices (either direction). The resulting minimum sample size per group was 60.

### 3.3. Gender Differences

The study composition is shown in Table 1 above, stratified by gender with respect to the other covariates. We found no significant difference between the mean age and/or distribution between the two genders. Likewise, we saw no difference between the groups with respect to the device used for OCT measurement. When comparing refractive errors, we observed a higher rate of hypermetropia among males (OR = 2.96, CI 95% 1.7–5.15, *p* < 0.001) and a higher rate of myopia among females (OR = 2.9, CI 95% 1.347–6.245, *p* = 0.005), while astigmatism incidence showed no propensity for gender.

**Figure 2:**
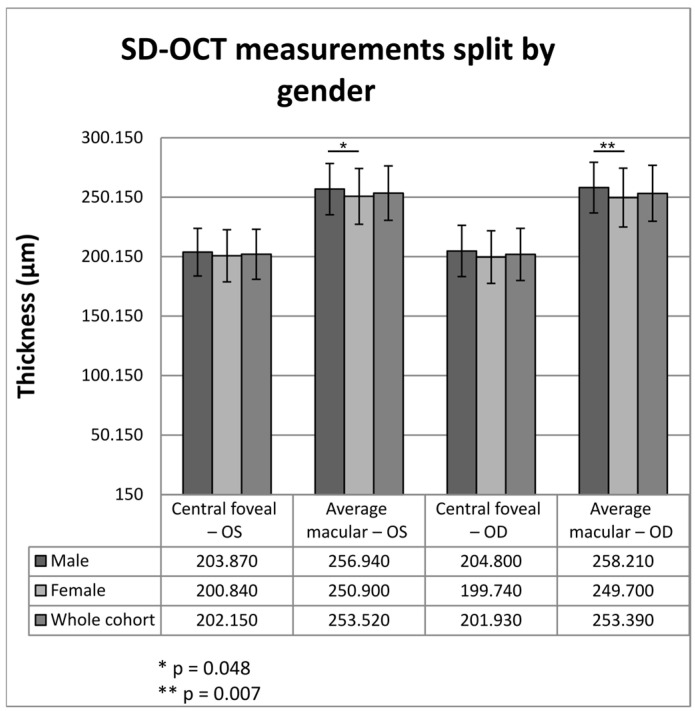
SD-OCT measurements based on gender.

When comparing the SD-OCT values, we found significant differences between genders (thicker in males) for average macular thickness in both eyes (*p* = 0.048 for the left eye and *p* = 0.007 for the right eye), while the central foveal thickness differences did not pass the significance threshold (*p* = 0.288 for the left eye and *p* = 0.084 for the right eye), although they also showed a trend for slightly higher values in males. The actual differences are illustrated in Figure 2.

### 3.4. Age

**Figure 3:**
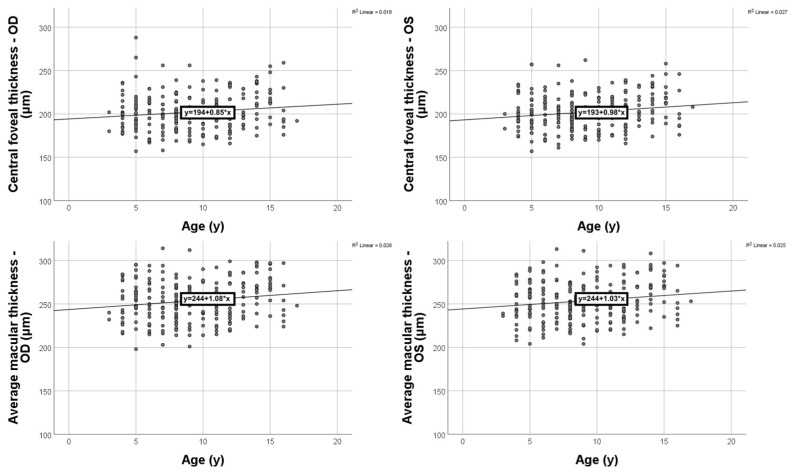
Age influence on SD-OCT measurements.

We assessed the dependence of the four OCT measurements on patient age, and we found weak positive relationships for each of them (three out of four passing the statistical significance threshold). The results are as follows (Figure 3): central foveal thickness (left eye), rho = 0.149, *p* = 0.025; average macular thickness (left eye), rho = 0.136, *p* = 0.041; central foveal thickness (right eye), rho = 0.118, *p* = 0.076; average macular thickness (right eye), rho = 0.142, *p* = 0.032.

### 3.5. Different Results, Different Machines

**Figure 4:**
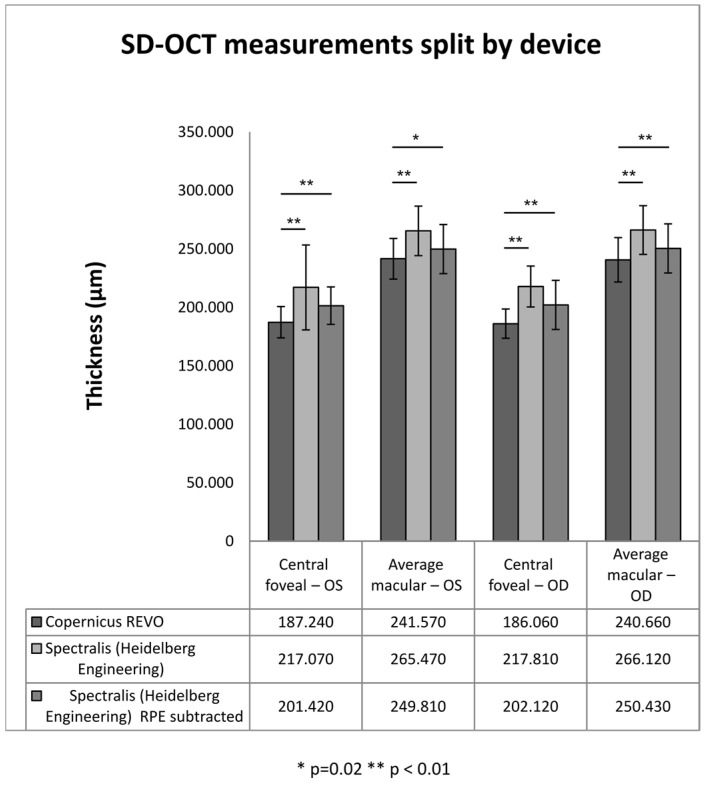
SD-OCT measurements between the two measuring devices. * *p* = 0.02, ** *p* < 0.01.

We compared the two measurement devices. Table 2 shows the patient distribution between devices with respect to age and refractive errors. The two samples were statistically equivalent in terms of age and refractive errors. As shown in Figure 4, statistically significant differences were identified between the devices for both macular and foveal measurements. These differences are maintained in each age group (Table 3).

The two OCT platforms employ different layer-based segmentation protocols for retinal thickness quantification: the Heidelberg system defines the outer retinal boundary at Bruch’s membrane, while the Revo system uses the inner border of the retinal pigment epithelium. Given this observation, and the significant differences between the two devices, we explored a method to obtain more similar results. For the Spectralis group, we measured RPE thickness and subtracted the values from the total retina thickness in each patient. We still found significant differences between the two devices with respect to the OCT measurements, albeit at a smaller margin. Figure 4 and Table 4 illustrate this.

Given the fact that significant differences are still noted, and considering the additional time needed in the clinical setting to perform the RPE subtraction, which cannot be automated in the examination protocol, we consider in unlikely to be applied daily by clinicians; therefore, we continued our analysis based on the default measurements.

### 3.6. Refraction Influence on Macular Thickness

When assessing for refractive error influence on SD-OCT measurements, we found no significant differences between groups with/without given refractive error (Table 5, Table 6 and Table 7).

## 4. Discussion

This study had multiple objectives. The major one was to establish normative data, dedicated to each device, given the previously reported variability in absolute measurements among the OCT devices. This study also had minor clinically relevant objectives, derived from the availability of the study cohorts: first, we aimed to see if we can interchange these two devices; for example, if a patient is examined with one device and, following treatment, is examined with the another device; second, we wanted to determine if there are patient-dependant factors influencing the measured values so that a single measurement in one patient can be contextualized not only by age but also by gender and refractive error. Our findings revealed distinct gender-related variations, with males generally demonstrating slightly greater macular thickness values. Although this trend was consistent across the two devices, the absolute macular thickness measurements differed significantly between the Spectralis SD-OCT and the Copernicus REVO SD-OCT. A weak positive relationship was observed between age and macular thickness. These values represent a valuable normative database for the Romanian paediatric population and support a more accurate interpretation of macular OCT findings in clinical and research settings.

Our analysis relating to macular thickness is anchored within the context established by the systematic review of paediatric OCT data conducted by Banc and Ungureanu [14]. This study underscores the necessity of analysing parameters separately for children under and over five years of age, due to the most significant morphological development occurring in early childhood. While many of the reviewed studies found no significant correlation between age and overall macular thickness in the broader paediatric population [15,16,17,18,19,20,21], this early developmental thickening, including a logarithmic increase in the photoreceptor layer, highlights the need for age-stratified reference data. An extensive analysis conducted by Song et al. [19] confirmed that macular thickness depends on age, sex, and axial length. Specifically, this adult-based study indicates that increasing age leads to a decline in the average inner, outer, and overall macular thickness, as well as macular volume (*p* ≤ 0.002). This age-dependent thinning correlates with histologic studies reporting age-related decreases in photoreceptor, ganglion cell, and retinal pigment epithelial cell densities [22,23] and reinforces the need for establishing paediatric, age-stratified normative values. Understanding the extent of physiological thinning with age is essential for identifying true abnormalities in younger populations, particularly since rapid macular maturation occurs in early life.

Regarding gender, Banc and Ungureanu [14] concluded that central macular OCT parameters demonstrated a tendency toward higher values in boys. Several studies reported that boys exhibited greater thickness values across various macular parameters, including the central macula, inner ring, and specific outer quadrants [24,25,26,27,28]. Most importantly, no studies were found to report greater overall macular OCT parameter values in girls. Song et al. [19], whose study population was composed of adults, analysed 198 individuals (mean age 55.6 ± 16.4 years, range 17–83) and similarly found that female subjects had consistently lower central subfield thickness (CST), average inner macular thickness, and overall macular volume than males, which aligns with our study and with the observations in many paediatric cohorts and supports the need for gender-specific data stratification.

Our findings regarding gender effects on macular thickness are directly comparable to recent paediatric normative studies. In a Saudi cohort of 135 healthy children (4–18 y), Raffa et al. [24] reported a mean macular thickness of 275.9 µm (SD: 17.7 µm) and demonstrated that boys had significantly thicker central macula than girls. Similarly, Jammal et al. [25], examining 144 Jordanian children (ages 6–16 years) using the Primus SD-OCT, found that boys had significantly higher CST than girls (adjusted mean 250.68 µm [95% CI: 246.68, 254.68] vs. 243.06 µm [95% CI: 239.29, 246.84]; *p* = 0.008). This male predominance aligns with our findings and demonstrates that gender-related differences in central macular parameters are observable in at least certain paediatric populations. However, this effect is not universally expressed across all populations. Wolf et al. [26], utilizing swept-source OCT (SS-OCT) in 55 healthy Swedish children (ages 4–16 years), established normative data for macular ganglion cell–inner plexiform layer (GCIPL) thickness with a mean average thickness of 68.0 µm (SD: 4.0 µm, range: 58–77 µm) and found no statistically significant correlations with gender (r = 0.02, *p* = 0.89). Additionally, Svensson et al. [27], utilizing Topcon DRI-Triton Plus SS-OCT in Swedish children (n = 69, ages 5–17 years), found that macular thickness did not differ appreciably between boys and girls; statistical testing showed no significant gender effect for total macular thickness (*p* = 0.06) or macular ganglion cell layer (GCL) thickness (*p* = 0.30). The observed discrepancy between populations suggests that gender effects on macular thickness are population-specific rather than universal features of paediatric macular development.

Beyond gender, our study’s findings regarding the influence of age of macular thickness are generally consistent with recent literature. Jammal et al. [25] reported that none of the macular parameters correlated with age in their paediatric cohort. Similarly, Raffa et al. [24], except for the superior inner macula. Runge et al. [28] examined a German paediatric cohort (n = 72, ages 4–17 years) using SD-OCT and reported that total macular volume (TMV) did not show a significant correlation with age (r = 0.14, *p* = 0.14). While TMV is a volumetric measure, this result aligns with findings indicating that mean macular thickness generally stabilizes after early childhood. Wolf et al. [26] found no significant correlation between age and GCIPL thickness (r = 0.12, *p* = 0.37). Svensson et al. [27] observed a mean total macular thickness of 287.5 ± 11.1 µm (5–7 years) and 290.5 ± 13.8 µm (8–17 years), with no significant differences by age (*p* = 0.32). These consistent findings, along with our observation of a weak positive relationship with age, suggest that once the critical period of early macular development is complete (by age of 5, as emphasized by Banc and Ungureanu [14]), macular thickness remains largely stable throughout childhood and adolescence. Importantly, this relative age independence provides a useful clinical context for interpreting gender-related differences, as observed gender effects cannot be attributed to differential age distributions between comparison groups.

An additional physiological factor known to influence macular thickness in paediatric populations is axial length (AL). Several studies have demonstrated that increased AL, particularly in myopic eyes, is associated with a reduction in central and parafoveal macular thickness [29,30] due to progressive elongation of the posterior segment of the globe, which results in mechanical stretching of the retina. Jin et al. [31] further suggested that myopia-associated retinal thinning may not reflect active thinning during adolescence, but rather an insufficient increase in retinal thickness during early childhood, when normal retinal growth should occur. This relationship has been reported in both paediatric and adult populations [32], underscoring the importance of AL as a confounding factor when interpreting macular OCT measurements. AL, a key determinant of macular thickness, was not measured in our study. Although we excluded high refractive errors and applied a small-eye length setting during OCT acquisition, these do not replace AL measurement, which is essential for accurately accounting for anatomical variability and represents a limitation of our study.

The geographic variation in gender-related macular thickness patterns warrants careful consideration. Studies from the Middle East consistently demonstrate male predominance in macular thickness. Jammal et al. in Jordan [25] and Raffa et al. in Saudi Arabia [24] observed significantly higher values in boys. In contrast, European studies present conflicting findings. Wolf et al. [26] found no gender effect on GCIPL thickness in Sweden. Svenson et al. [27] similarly reported that macular thickness values were not influenced by gender. This geographic divergence suggests that gender effects on macular thickness may be influenced by ethnic or genetic factors, with expression varying across populations. Such heterogeneity reinforces that sex-specific normative references should be population-based rather than globally applied. There is a gap in the current literature regarding macular thickness in Eastern European paediatric population; therefore, this study addresses this ethnically unique segment specifically and reveals its characteristic variability.

The establishment of normative reference ranges for macular thickness requires careful consideration of device-specific measurement characteristics. Nam et al. [33] conducted a comparison of retinal thickness measurements among four different OCT devices and identified significant differences in the reported thickness values, confirming that measurements obtained from different platforms cannot be used interchangeably.

Spectralis SD-OCT requires a signal strength of >20/40, while Copernicus REVO SD-OCT requires >7. This indicates that the two devices use different signal strength thresholds for image quality control. It is one of the reasons they have different measurements and it is a strong argument for consulting device dedicated normative data. Haffane et al. [34] critically illustrated that various OCT devices (Cirrus SD-OCT, Spectralis and RTVue 100 Avanti OCT devices) yield a distinct retinal nerve fibre layer (RNFL), GCL, and central macular thickness results, which are influenced by different algorithms, acquisition techniques, and calibration protocols, ultimately leading to non-comparable results when measuring macular thickness across devices. Similarly, Kola et al. [35] noted that macular thickness measurements from two different scan modes of the Optovue RTVue OCT device exhibited excellent repeatability but raised questions regarding their generalizability across other devices. This indicates that while repeatability may be high for a specific device, it does not imply uniformity across different systems; therefore, it is crucial to use the same device throughout the follow-up period of the patient. Although Hou et al. [36] demonstrated that there were no statistically significant measurement differences between Triton SS-OCT and Maestro SD-OCT, this is likely attributable to their use of similar software and algorithms, as well as minimal differences in axial resolution and pixel calibration factors. Nevertheless, these findings highlight the importance of developing device-specific normative data to ensure a reliable and comparable macular thickness assessment. Our analysis using two distinct SD-OCT platforms addresses this critical need for device-specific gender-stratified analysis within a single cohort.

The differences in macular thickness values observed between the Spectralis and Copernicus REVO in our own study can be explained by several technical distinctions between the two systems. The REVO’s superior axial resolution (3 µm versus Spectralis’ 3.9 µm) and higher scan speed (130,000 versus 85,000 A-scans/second) enables finer image capture along the depth axis and allows faster data acquisition. REVO’s AI DeNoise algorithms enhance single-frame tomograms to multi-scan quality, while the Spectralis relies on traditional frame-averaging methods, yielding different levels of noise reduction and image contrast. Furthermore, the REVO’s extended depth software (approximately 6 mm versus 1.9 mm) may improve posterior visualization. These systematic differences in image reconstruction, axial resolution, and scan speed represent some of the factors that contributed to the observed differences in macular retinal thickness between the devices [37,38]. We also noted a difference in the way total retina thickness is measured by the devices and proposed a method to attenuate the difference by measuring RPE thickness on the Spectralis and subtracting it from the total retina thickness measurement. However, the significant differences were maintained; therefore, we can conclude that clinicians should not use the devices interchangeably. In particular situations where they only have previous measurements from the Copernicus REVO device and want to compare them to Spectralis, they could spend some time performing the aforementioned steps so that the values are closer; however, they need to keep in mind that a difference around 10–15 μm is still to be expected.

An additional consideration in paediatric OCT imaging relates to patient cooperation and the presence of motion artefacts. Younger children often exhibit limited fixation, variable attention, and increased eye movements, all of which can affect the quality of the scans or lead to segmentation errors, measurement variability, and affect the reproducibility of the measurements. To minimize these effects, all OCT images were reviewed individually, and scans with poor image quality, motions artefacts, or obvious segmentations errors were excluded from the analysis. When necessary, multiple scans were acquired and the highest quality image was selected. Children were also encouraged to fixate on the internal target and verbally reassured to facilitate cooperation during image acquisition. Both devices used in this study incorporate an advanced eye-tracking system, which further reduce the likelihood of artefacts. Nonetheless, the influence of motion artefacts cannot be fully eliminated in paediatric imaging and represents a limitation of this study.

Study limitations include the relatively small sample size, the need to discard some of the measurements due to poor quality, mostly caused by the lack of patient compliance and the use of two separate groups of patients for each device. The latter led to an inability to use paired statistical tests, which was impossible to overcome due to administrative restrictions. The significant difference in myopia and hypermetropia between males and females (Table 1) may theoretically be a confounding factor in the gender differences observed [6]. However, Table 2 shows that the refraction defects do not significantly influence measurements in our devices. All examined children were Romanian, so we did not have ethnical differences to study.

## 5. Conclusions

This study provides gender-specific normative data for macular retinal thickness in Romanian children using two commonly employed SD-OCT devices. Boys demonstrated greater macular thickness than girls across both platforms. The significant differences between the Heidelberg and Copernicus SD-OCT device measurements confirm that the results obtained from different OCT systems are not interchangeable. These findings highlight the importance of using device- and gender-specific normative databases for accurate clinical interpretation and reliable comparison of paediatric OCT measurements. Moreover, they underscore the need for further studies in paediatric populations to expand and validate normative OCT databases, which are essential for improving diagnostic accuracy and supporting standardized clinical assessment in children.

## Figures and Tables

**Table 1:** Demographic and clinical characteristics of the study population, stratified by gender.

Variable	Number of Eyes (*n*)	Male	Female	Whole Cohort	*p*-Value
Age groups					0.552
1–3 years	4	1 (0.4%)	1 (0.4%)	2 (0.8%)	
4–6 years	122	28 (12.4%)	33 (14.6%)	61 (27.0%)	
7–9 years	132	32 (14.2%)	34 (15.0%)	66 (29.2%)	
10–12 years	106	17 (7.5%)	36 (15.9%)	53 (23.5%)	
13–15 years	72	17 (7.5%)	19 (8.4%)	36 (15.9%)	
16–18 years	16	3 (1.3%)	5 (2.2%)	8 (3.5%)	
Age (mean)		8.7 ± 3.48	9.38 ± 3.49	9.09 ± 3.50	0.149
Refraction groups					
Hypermetropia present	182	54 (23.9%)	37 (16.4%)	91 (40.3%)	<0.001 *
Myopia present	84	10 (4.4%)	32 (14.2%)	42 (18.6%)	0.005 *
Astigmatism present	48	9 (4.0%)	15 (6.6%)	24 (10.6%)	0.557
Device					0.591
Copernicus REVO	226	47 (20.8%)	66 (29.2%)	113 (50%)	
Spectralis	226	51 (22.6%)	62 (27.4%)	113 (50%)	

Note: *p*-values compare males vs. females. * Statistically significant (*p* < 0.05).

**Table 2:** Age and refractive error distribution between the two measuring devices.

Variable	Copernicus REVO	Spectralis (Heidelberg Engineering)	Whole Cohort	*p*-Value
Age (mean)	8.95 ± 3.319	9.23 ± 3.684	9.09 ± 3.501	0.647
Refraction groups				
Hypermetropia present	47 (20.8%)	44 (19.5%)	91 (40.3%)	0.684
Myopia present	24 (10.6%)	18 (8.0%)	42 (18.6%)	0.305
Astigmatism present	14 (6.2%)	10 (4.4%)	24 (10.6%)	0.388

Note: *p*-values compare Copernicus REVO vs. Spectralis (Heidelberg Engineering).

**Table 3:** SD-OCT measurements between the two measuring devices, stratified by age group.

Variable	Age Group	Number of Eyes (*n*)	Copernicus REVO	Spectralis (Heidelberg Engineering)	Whole Cohort	*p*-Value
Central foveal thickness—OS (μm)	1–3 years	2	183.00	200.00	191.50 ± 12.021	N/A
	4–6 years	61	188.10 ± 16.414	214.23 ± 17.345	201.38 ± 21.309	<0.001 *
	7–9 years	66	186.33 ± 12.574	213.27 ± 18.443	198.58 ± 20.482	<0.001 *
	10–12 years	53	183.68 ± 12.190	217.40 ± 11.690	199.58 ± 20.714	<0.001 *
	13–15 years	36	195.15 ± 9.797	224.96 ± 13.626	214.19 ± 18.984	<0.001 *
	16–18 years	8	188.80 ± 9.203	227.00 ± 19.000	203.13 ± 23.290	0.008 *
Average macular thickness—OS (μm)	1–3 years	2	236.00	239.00	237.50 ± 2.121	N/A
	4–6 years	61	243.63 ± 22.850	260.81 ± 21.617	252.36 ± 23.684	0.004 *
	7–9 years	66	241.44 ± 16.326	260.00 ± 24.741	249.88 ± 22.435	0.001 *
	10–12 years	53	237.82 ± 14.601	266.08 ± 18.039	251.15 ± 21.534	<0.001 *
	13–15 years	36	246.92 ± 14.517	278.70 ± 13.326	267.22 ± 20.578	<0.001 *
	16–18 years	8	238.20 ± 10.281	270.67 ± 21.079	250.38 ± 21.672	0.024 *
Central foveal thickness—OD (μm)	1–3 years	2	180.00	202.00	191.00 ± 15.556	N/A
	4–6 years	61	186.60 ± 15.201	216.32 ± 21.043	201.70 ± 23.613	<0.001 *
	7–9 years	66	186.03 ± 12.369	214.27 ± 16.519	198.86 ± 20.122	<0.001 *
	10–12 years	53	182.11 ± 10.236	216.24 ± 13.355	198.21 ± 20.800	<0.001 *
	13–15 years	36	192.38 ± 9.483	225.61 ± 13.439	213.61 ± 20.157	<0.001 *
	16–18 years	8	190.00 ± 10.416	227.00 ± 33.601	203.88 ± 27.409	0.054
Average macular thickness—OD (μm)	1–3 years	2	232.00	240.00	236.00 ± 5.657	N/A
	4–6 years	61	242.70 ± 22.533	261.90 ± 21.115	252.46 ± 23.707	0.001 *
	7–9 years	66	240.19 ± 20.067	260.30 ± 24.071	249.33 ± 24.023	<0.001 *
	10–12 years	53	235.43 ± 14.294	267.64 ± 16.800	250.62 ± 22.358	<0.001 *
	13–15 years	36	250.38 ± 16.870	278.13 ± 14.303	268.11 ± 20.220	<0.001 *
	16–18 years	8	237.60 ± 11.632	272.00 ± 24.515	250.50 ± 23.791	0.033 *

Note: *p*-values compare Copernicus REVO vs. Spectralis (Heidelberg Engineering). * Statistically significant (*p* < 0.05).

**Table 4:** SD-OCT measurements between the two measuring devices, stratified by age group after subtracting RPE thickness in Spectralis.

Variable	Age Group	Number of Eyes (*n*)	Copernicus REVO	Spectralis (Heidelberg Engineering) (RPE Subtracted)	Whole Subgroup	*p*-Value
Central foveal thickness—OS (μm)	1–3 years	2	183.00	185.00	184.00 ± 1.414	N/A
	4–6 years	61	188.10 ± 16.414	198.58 ± 17.355	193.43 ± 17.570	0.019 *
	7–9 years	66	186.33 ± 12.574	198.07 ± 18.560	191.67 ± 16.537	0.003 *
	10–12 years	53	183.68 ± 12.190	201.64 ± 11.856	192.15 ± 14.966	<0.001 *
	13–15 years	36	195.15 ± 9.797	208.74 ± 13.575	203.83 ± 13.876	0.003 *
	16–18 years	8	188.80 ± 9.203	211.67 ± 19.009	197.38 ± 17.079	0.057
Average macular thickness—OS (μm)	1–3 years	2	236.00	224.00	230.00 ± 8.485	N/A
	4–6 years	61	243.63 ± 22.850	245.16 ± 21.666	244.41 ± 22.083	0.79
	7–9 years	66	241.44 ± 16.326	244.80 ± 24.859	242.97 ± 20.544	0.529
	10–12 years	53	237.82 ± 14.601	250.32 ± 17.627	243.72 ± 17.140	0.007 *
	13–15 years	36	246.92 ± 14.517	262.48 ± 13.080	256.86 ± 15.402	0.002 *
	16–18 years	8	238.20 ± 10.281	255.33 ± 21.221	244.63 ± 16.361	0.165
Central foveal thickness—OD (μm)	1–3 years	2	180.00	187.00	183.50 ± 4.950	N/A
	4–6 years	61	186.60 ± 15.201	201.00 ± 21.139	193.92 ± 19.693	0.003 *
	7–9 years	66	186.03 ± 12.369	198.77 ± 16.126	191.82 ± 15.468	0.001 *
	10–12 years	53	182.11 ± 10.236	200.00 ± 13.401	190.55 ± 14.785	<0.001 *
	13–15 years	36	192.38 ± 9.483	209.65 ± 13.320	203.42 ± 14.598	<0.001 *
	16–18 years	8	190.00 ± 10.416	212.00 ± 32.047	198.25 ± 22.024	0.19
Average macular thickness—OD (μm)	1–3 years	2	232.00	225.00	228.50 ± 4.950	N/A
	4–6 years	61	242.70 ± 22.533	246.58 ± 21.427	244.67 ± 21.881	0.493
	7–9 years	66	240.19 ± 20.067	244.80 ± 23.675	242.29 ± 21.731	0.395
	10–12 years	53	235.43 ± 14.294	251.40 ± 16.348	242.96 ± 17.153	<0.001 *
	13–15 years	36	250.38 ± 16.870	262.17 ± 14.151	257.92 ± 16.013	0.032 *
	16–18 years	8	237.60 ± 11.632	257.00 ± 23.065	244.88 ± 18.169	0.155

Note: *p*-values compare Copernicus REVO vs. Spectralis (Heidelberg Engineering). * Statistically significant (*p* < 0.05).

**Table 5:** Hypermetropia influence on SD-OCT measurements.

Variable	Without Hypermetropia	With Hypermetropia	Whole Cohort	*p*-Value
Central foveal thickness—OS (μm)	203.04 ± 20.725	200.84 ± 21.665	202.15 ± 21.089	0.445
Average macular thickness—OS (μm)	253.46 ± 22.193	253.60 ± 23.852	253.52 ± 22.823	0.963
Central foveal thickness—OD (μm)	202.25 ± 21.516	201.46 ± 22.721	201.93 ± 21.963	0.794
Average macular thickness—OD (μm)	253.13 ± 23.528	253.78 ± 23.885	253.39 ± 23.622	0.841

Note: *p*-values compare participants with vs. without hypermetropia.

**Table 6:** Myopia influence on SD-OCT measurements.

Variable	Without Myopia	With Myopia	Whole Cohort	*p*-Value
Central foveal thickness—OS (μm)	201.77 ± 21.566	203.83 ± 19.006	202.15 ± 21.089	0.569
Average macular thickness—OS (μm)	253.04 ± 23.280	255.62 ± 20.834	253.52 ± 22.823	0.510
Central foveal thickness—OD (μm)	201.55 ± 22.177	203.60 ± 21.175	201.93 ± 21.963	0.578
Average macular thickness—OD (μm)	252.93 ± 24.258	255.40 ± 20.749	253.39 ± 23.622	0.542

Note: *p*-values compare participants with vs. without myopia.

**Table 7:** Astigmatism influence on SD-OCT measurements.

Variable	Without Astigmatism	With Astigmatism	Whole Cohort	*p*-Value
Central foveal thickness—OS (μm)	202.24 ± 20.991	201.46 ± 22.347	202.15 ± 21.089	0.872
Average macular thickness—OS (μm)	253.78 ± 22.824	251.29 ± 23.179	253.52 ± 22.823	0.614
Central foveal thickness—OD (μm)	202.06 ± 21.994	200.88 ± 22.131	201.93 ± 21.963	0.806
Average macular thickness—OD (μm)	253.80 ± 23.554	249.96 ± 24.425	253.39 ± 23.622	0.470

Note: *p*-values compare participants with vs. without astigmatism.

## Data Availability

The data presented in this study are available on request from the corresponding author.

## Abbreviations

The following abbreviations are used in this manuscript:

OCT	Optical coherence tomography
SD-OCT	Spectral-domain OCT
TD-OCT	Time-domain OCT
BCVA	Best-corrected visual acuity
SE	Spherical equivalent
D	Dioptres
VA	Visual acuity
SD	Standard deviation
MDE	Minimum detectable effect
OD	Odds ratio
CI	Confidence interval
CST	Central subfield thickness
SS-OCT	Swept source OCT
GCIPL	Ganglion cell–inner plexiform layer
GCL	Ganglion cell layer
TMV	Total macular volume
RNFL	Retinal nerve fibre layer
